# Hide and Seek: Intermittent Preexcitation Wolff-Parkinson-White Syndrome Case Report and Management Overview

**DOI:** 10.7759/cureus.8971

**Published:** 2020-07-02

**Authors:** Neil P Larson, Jennifer B Rosenthal, Rachel E Bridwell, Lloyd Tannenbaum, Amber Cibrario

**Affiliations:** 1 Emergency Medicine, Brooke Army Medical Center, Fort Sam Houston, San Antonio, USA; 2 Psychiatry, University of Texas Health Science Center at San Antonio, San Antonio, USA; 3 Emergency Medicine, Brooke Army Medical Center, Fort Sam Houston, USA

**Keywords:** wolff-parkinson-white, supraventricular tachycardia, intermittent preexcitation

## Abstract

Wolff-Parkinson-White (WPW) syndrome is an uncommon form of cardiac preexcitation due to an underlying structural accessory pathway, which may lead to potentially lethal arrhythmias. Classic electrocardiogram (ECG) findings of WPW include short PR interval, slurred upstroke of the QRS complex, and prolonged QRS duration. However, in intermittent preexcitation, a rare variant in contrast to continuous preexcitation, these findings are not always present, thus masking a diagnosis of WPW syndrome. Consequently, this may adversely affect or delay the appropriate treatment of short-term tachyarrhythmias and long-term definitive therapies for this syndrome. The emergency physician should promptly obtain an ECG after the termination of any tachyarrhythmia, and maintain a high index of suspicion for intermittent preexcitation with typical WPW ECG findings which were not present on prior studies. The authors present a case of a 17-year-old female diagnosed with an intermittent preexcitation variant of WPW syndrome after a case of successfully treated symptomatic supraventricular tachycardia (SVT).

## Introduction

Wolff-Parkinson-White (WPW) syndrome is a form of electrical preexcitation caused by a congenital accessory pathway known as the “Bundle of Kent”, which bypasses normal atrioventricular (AV) nodal and His Purkinje conduction system regulation. With a predilection for males most commonly presenting in the third decade of life, this preexcitation syndrome may lead to potentially lethal arrhythmias due to unregulated electrical impulse conduction between the cardiac atria and ventricles [1,2]. Classic ECG findings of WPW include a shortened PR interval of less than 120 milliseconds, a slurred upstroke of the QRS complex referred to as the delta wave, and a widened QRS greater than 110 milliseconds [3]. This ECG pattern has been estimated at one to three individuals per 1,000 persons; however, the pattern alone is far more common in asymptomatic individuals than those with associated cardiac arrhythmias secondary to the accessory pathway, marking the diagnosis of true WPW syndrome [2]. One cohort study revealed a rate of tachyarrhythmias as 1.0% per year of individuals with WPW pattern [4]. In the pediatric population of 6 to 20 years old, the prevalence of WPW syndrome was less than 0.07% [5]. While atrioventricular reentrant tachycardia (AVRT) may comprise the majority of tachyarrhythmias in WPW, atrial fibrillation (AF) and atrial flutter occur in approximately 20% and 7% of WPW patients, respectively [3].

## Case presentation

**Figure 1:**
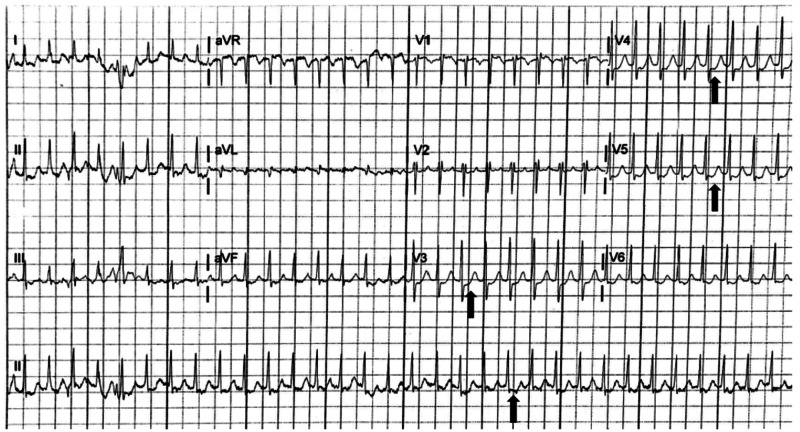
Initial electrocardiogram demonstrating supraventricular tachycardia with diffuse ST segment depressions (black arrows)

**Figure 2:**
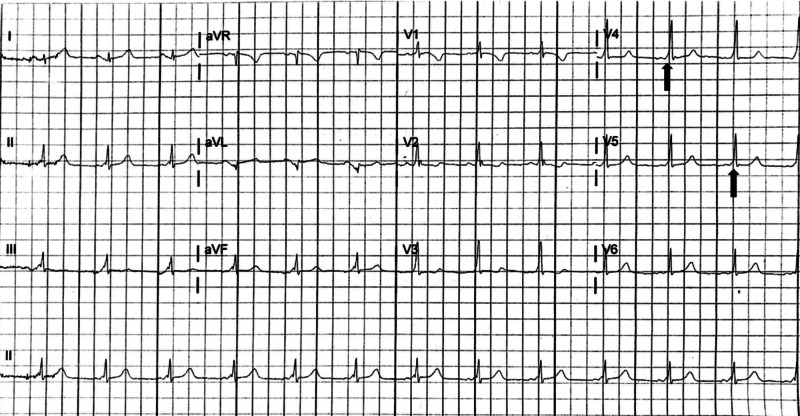
Electrocardiogram obtained after resolution of supraventricular tachycardia, demonstrating normal sinus rhythm with short PR interval, wide QRS duration, and delta waves (black arrows)

**Figure 3:**
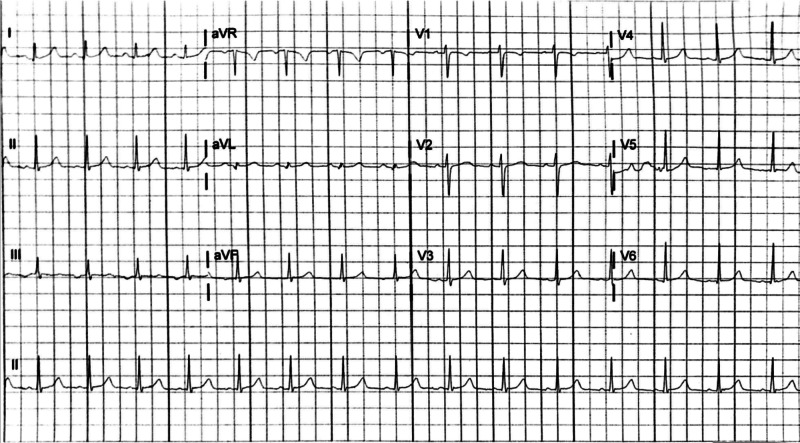
Electrocardiogram obtained after resolution of initial episode of supraventricular tachycardia two months prior, demonstrating normal sinus rhythm with intervals within normal limits

A 17-year-old female with past medical history of generalized anxiety disorder (GAD) and one episode of SVT two months earlier presented to the emergency department with a chief complaint of palpitations at rest. Initial vital signs included heart rate of 200 beats per minute, blood pressure of 97/63 mm Hg, 18 breaths per minute (bpm) and oxygen saturation of 95% on room air. While the patient appeared uncomfortable, she was speaking in full sentences and reported that symptoms were consistent with her previous episode of SVT. An ECG confirmed SVT (Figure 1). After unsuccessful vagal maneuvers, 6 mg of IV adenosine was rapidly administered, followed by rapid administration of 10 milliliters of 0.9% normal saline, demonstrating successful conversion to sinus rhythm on repeat ECG (Figure 2). However, this ECG revealed WPW morphology including short PR interval (100 milliseconds), delta wave, and mildly prolonged QRS (117 milliseconds). The patient reported resolution of symptoms, and repeat vital signs were normal. Laboratory values and chest radiograph were unremarkable. Given the concern for a new diagnosis of WPW, pediatric cardiology was consulted and confirmed the new diagnosis of WPW. Upon a more thorough review of the patient's medical records, an ECG obtained after the termination of her initial episode of SVT from two months after prior was retrieved which did not show evidence of WPW, supporting the diagnosis of intermittent preexcitation (Figure 3). The patient was prescribed oral atenolol at 25 milligrams twice daily in conjunction with short-term pediatric cardiology clinic follow-up, and did not return to the emergency department within the next 30 days. Due to the side effects from atenolol, the patient and her family elected to discontinue the medication and instead, opted for cardiac catheter ablation.

## Discussion

Due to temporary accessory pathway activation, the true prevalence of intermittent preexcitation in WPW syndrome may be underreported as a result of protean ECG evidence supportive of the diagnosis. Multiple studies have suggested an approximate 13% prevalence of intermittent preexcitation in the pediatric WPW syndrome population [6,7]. Orthodromic AVRT is a particular subtype of SVT accounting for up to 95% of reentrant tachycardias in WPW patients, caused by anterograde conduction along the AV node with retrograde conduction along the accessory pathway [8,9]. Orthodromic AVRT and other forms of SVT including atrioventricular nodal reentrant tachycardia (AVNRT) share many electrocardiographic characteristics. While electrophysiologic studies (EPS) are required to definitively distinguish AVRT from AVNRT, ECG markers which may indicate orthodromic AVRT include a heart rate typically between 200 and 300 beats per minute, QRS duration of less than 120 milliseconds, ST depressions, T wave inversions, and retrograde P waves [3]. Figure 1 displays a rate of nearly 200 beats per minute, a narrow QRS complex, and ST segment depressions, though these may also be demonstrated in typical SVT rhythms. Similarly, ST depressions reflect demand ischemia at these rapid heart rates, which further adds difficulty in attempting to differentiate orthodromic AVRT from other forms of SVT. While immediate electrical cardioversion is indicated in any hemodynamically unstable patient in acute arrhythmia, adenosine has been demonstrated to be safe and effective for the treatment of otherwise stable patients with multiple forms of SVT, including orthodromic AVRT with WPW syndrome [10,11]. Thus, if vagal maneuvers are unsuccessful, rapid administration of IV adenosine remains first-line medication treatment for termination of this arrhythmia [10]. After termination of the arrhythmia, the typical ECG characteristics were apparent, likely due to residual preexcitation. While the time between the termination of the patient’s first episode of SVT and her repeat normal ECG is not known, minimizing the delay between SVT termination and repeat ECG may have been essential to make the intermittent preexcitation WPW diagnosis. Fortunately, patients with intermittent preexcitation have less frequent episodes of SVT over time in comparison with those with continuous preexcitation [4].

A myriad of other tachyarrhythmias may occur in WPW syndrome. Far less common than orthodromic AVRT, antidromic AVRT may also occur at an incidence of 5% of WPW patients [3]. Procainamide, a class 1A antiarrhythmic medication that acts by inhibiting cardiac myocyte sodium channels, is an appropriate medical treatment option in otherwise stable antidromic AVRT patients, though electrical cardioversion stands as another reasonable treatment option [3,12]. Rarely, WPW may lead to sudden cardiac death (SCD) due to atrial fibrillation and subsequent ventricular tachycardia and ventricular fibrillation, accounting for 0.0%-0.6% deaths per WPW patient per year [4]. Fortunately, in intermittent preexcitation, patients generally have a longer refractory period and a decreased conductive accessory pathway between the atria and the ventricles [13]. This is considered a relatively protective factor, as AF is less likely to initiate a sustained ventricular tachycardia, subsequent ventricular fibrillation with resulting SCD [4]. However, administering AV nodal blocking agents such as adenosine and verapamil during AF in WPW is contraindicated, as this may preferentially direct the current through the accessory pathway, causing iatrogenic decompensation. Procainamide, as previously mentioned, is the first-line medical therapy for stable patients with ibutilide, a class 3 antiarrhythmic medication that primarily inhibits potassium channels, being a reasonable alternative [3,12]. However, electrical cardioversion may ultimately be warranted [3,12]. Even in an otherwise stable WPW patient with a tachyarrhythmia of uncertain rhythm, synchronized cardioversion may be an appropriate, and potentially safest treatment option.

Patients with a suspected diagnosis of WPW necessitate cardiology referral. Specifically, electrophysiology cardiology physicians may confirm the diagnosis of an accessory pathway and strategize future treatments through EPS. According to 2015 expert guidelines, catheter ablation, a procedure performed by electrophysiology cardiologists, is first-line treatment for individuals who have ever experienced symptomatic SVT to include orthodromic AVRT, as this definitive therapy is both safe and effective [10]. Alternatively, a number of medical therapies, including beta-blockers are available to suppress episodes of SVT including orthodromic AVRT [10]. In the emergency department setting, after the termination of an acute tachyarrhythmia, certain asymptomatic and hemodynamically stable patients may be discharged from the emergency department after otherwise reassuring workup and short term observation. However, this decision should be made in conjunction with cardiology to not only ensure optimal disposition, but also to expedite needed inpatient or outpatient testing and treatment.

## Conclusions

The emergency physician should suspect a diagnosis of intermittent preexcitation with characteristic WPW findings on ECG not appreciated on prior studies in patients who have experienced tachyarrhythmias. While difficult to diagnose given the very low prevalence and fleeting ECG evidence of an accessory pathway, promptly obtaining ECG studies after acute arrhythmia termination may not only establish a diagnosis of intermittent preexcitation variant of WPW but also guide both short and long term therapy. Though intermittent preexcitation is less predisposing to both benign and malignant arrhythmias in comparison to continuous preexcitation, consultation with cardiology in the emergency department is appropriate for both disposition and treatment planning.
